# Hydroxychloroquine therapy for women with both recurrent pregnancy loss and autoimmune disease: association with pregnancy outcomes and maternal-fetal complications

**DOI:** 10.3389/fimmu.2026.1756063

**Published:** 2026-02-10

**Authors:** Mengyang Du, Ruixiu Zhang, Yu Na, Liying Peng, Shihua Bao

**Affiliations:** Department of Reproductive Immunology, Shanghai Key Laboratory of Maternal-Fetal Medicine, Shanghai Institute of Maternal-Fetal Medicine and Gynecologic Oncology, Shanghai First Maternity and Infant Hospital, School of Medicine, Tongji University, Shanghai, China

**Keywords:** autoimmune disease, hydroxychloroquine (HCQ), preterm birth, recurrent pregnancy, loss, maternal-fetal complications

## Abstract

**Objective:**

To evaluate the impact of hydroxychloroquine (HCQ) on pregnancy outcomes and maternal and fetal complications in women with a history of recurrent pregnancy loss (RPL) and autoimmune disease.

**Methods:**

The medical records of pregnant women with a history of RPL and autoimmune disease attending the Shanghai First Maternity and Infant Hospital between January 2017 and December 2019 were retrospectively reviewed. Primary outcomes were gestational week at delivery (term: ≥ 37 weeks; preterm: < 37 weeks). Secondary outcomes were mid-to-late pregnancy loss and maternal and fetal complications, defined in accordance with national and international guidelines.

**Results:**

103 (20.9%) patients were exposed to HCQ (0.1g twice daily), 389 (79.1%) were not. There were 436 full-term births, 48 preterm births, 5 late-term miscarriages, 2 stillbirths, and 1 fetal malformation-induced abortion. Univariable/multivariable analyses showed a potential protective trend of HCQ against preterm birth (unadjusted OR 0.86 [95% CI 0.40–1.84], p=0.70; Model 1 [adjusted for age, body mass index (BMI), recurrent abortion, antiphospholipid antibodies, undifferentiated connective tissue disease (UCTD)/Sjogren’s syndrome (SS), serological markers]: OR 0.59 [95% CI 0.19–1.84], p=0.37; Model 2 [additional adjustment for low molecular weight heparin (LMWH), intravenous immunoglobulin (IVIG), prednisone, aspirin]: OR 0.41 [95% CI 0.11–1.51], p=0.18). Fetal weight (HCQ: 3215.46 ± 459.21g vs. non-HCQ: 3226.96 ± 500.01g, p=0.84) and maternal-fetal complications showed no differences. Subgroup analysis stratified by autoimmune diagnosis (antiphospholipid syndrome [APS], UCTD, SS) revealed no heterogeneity (APS: OR 0.78 [95% CI 0.28, 2.22], p = 0.64; UCTD: OR 0.52 [95% CI 0.15, 1.81], p = 0.30; SS: OR 1.03 [95% CI 0.12, 8.87], p = 0.98).

**Conclusion:**

This study showed that in women with a history of RPL and autoimmune disease, HCQ use was not statistically significantly associated with adverse pregnancy outcomes or maternal-fetal complications. A potential protective trend against preterm birth was observed, but this association did not reach statistical significance.

## Introduction

Recurrent pregnancy loss (RPL), defined as the occurrence of ≥ 2 miscarriages, affects an estimated 2% of fertile couples worldwide (1). The etiology of RPL is multifactorial and may be unexplained in approximately 75% of patients (2). Autoimmune factors may contribute to an estimated 20% of RPL cases (3), with autoimmune diseases such as antiphospholipid syndrome (APS), Sjogren’s syndrome (SS), and undifferentiated connective tissue disease (UCTD) being associated with RPL (4–7). These diseases generate autoantibodies that cause coagulation and immune dysfunction, ultimately resulting in pregnancy failure.

An optimal treatment strategy for managing RPL in women with autoimmune disease has yet to be established (4, 8, 9). Currently, anticoagulants (e.g., low molecular weight heparin [LMWH] or unfractionated heparin [UFH]), nonsteroidal anti-inflammatory drugs (e.g. aspirin), immunotherapies (e.g., prednisone, cyclosporine-A), hydroxychloroquine (HCQ), and supplements (e.g., folic acid, calcium, Vitamin D) may be used to manage pregnant women with autoimmune disease (10, 11). These therapies are used to treat maternal disease and may prevent pregnancy losses (12).

Hydroxychloroquine (HCQ) is an antimalarial medication widely used in the treatment of systemic lupus erythematosus (SLE), and other rheumatic disorders, and beyond its approved indications in other autoimmune diseases such as SS (13). HCQ exerts an immunosuppressive effect through the inhibition of antigen presentation and Toll-like receptor activity, reduces thrombosis, and alleviates inflammatory reactions (13, 14). During pregnancy, HCQ is effective for mitigating the activity of autoimmune disease, while the immunomodulatory, anti-thrombotic, vascular-protective and anti-infectious properties of HCQ may protect against RPL (15). Evidence suggests that HCQ can improve pregnancy outcomes in patients with refractory obstetric APS and can reduce placental inflammation and improve trophoblast function, thereby decreasing placental insufficiency and enhancing maternal-fetal health in high-risk pregnancies (16–18).

Since HCQ is widely used for autoimmune diseases in women of childbearing age, including during pregnancy, and can cross the placental barrier with similar concentrations in umbilical blood and maternal blood, concerns have been raised about potential teratogenic effects of HCQ on the fetus and the risk of adverse pregnancy outcomes (19). Although most studies have shown that HCQ is safe in pregnant women with autoimmune diseases, conclusions can vary (20, 21). Recently, a population-based cohort study of HCQ-exposed or non-exposed pregnancies in the United States reported an increased risk for major congenital malformations associated with first-trimester HCQ use (22). In contrast, a population-based cohort study of all singleton births (2006–2021) among individuals with prevalent SLE or rheumatoid arthritis (RA) in Sweden reported no teratogenic effects of HCQ exposure during the 3 months preceding pregnancy and the first trimester (23).

As most previous studies were underpowered or focused solely on APS (20, 21, 24), there remains an unmet need for more research to fully understand the impact that HCQ may have on pregnancy outcomes among women with autoimmune diseases. In particular, studies reporting the effects of HCQ on fetal development and pregnancy outcomes in patients with RPL and various autoimmune diseases in China are limited, and there are a lack of analyses clarifying the safety of HCQ by type of autoimmune disease. The objective of this study was to evaluate the impact of HCQ on mid-late pregnancy outcomes and maternal and fetal complications in patients with a history of RPL and autoimmune disease in Shanghai.

## Methods

### Study population

Pregnant women with a history of RPL and autoimmune disease attending the Shanghai First Maternity and Infant Hospital between January 2017 and December 2019 were eligible for this retrospective single-center cohort study.

Inclusion criteria were: 1) singleton pregnancy (spontaneous conception); 2) normal nuchal translucency (NT) scan at 12 weeks’ gestation; 3) history of RPL (≥2 consecutive spontaneous miscarriages); and 4) autoimmune disease (APS, UCTD, SS) diagnosed according to international criteria (e.g., APS: Sydney criteria 2006; SS: ACR/EULAR criteria 2016) combining clinical symptoms and serological biomarkers (e.g., antiphospholipid antibodies, antinuclear antibodies).

Exclusion criteria were: 1) spontaneous pregnancy loss before 12 weeks’ gestation; 2) abnormal NT scan at 12 weeks’ gestation; 3) no indication of autoimmune disease; 4) missing demographic and/or clinical information; 5) multiple gestation pregnancy; or 6) pregnancy conceived by *in vitro* fertilization (IVF).

### Data collection

Medical records of included patients were reviewed, and relevant data were collected. Baseline demographic and clinical characteristics included age, pre-pregnancy body mass index (BMI), pre-pregnancy serology test results, history of and number of prior miscarriages, history of and number of previous IVF failures, and type of autoimmune disease. Pregnancy outcomes included pregnancy loss in mid-to-late pregnancy, gestational age at delivery, birth weight, and Apgar score. Maternal and fetal complications were recorded for singleton pregnancies. Medication utilization during pregnancy included HCQ use, determined from prescription records indicating HCQ dispensing at any point during gestation, and use of other immunosuppressive agents (prednisone, cyclosporin A), replacement therapy (intravenous immunoglobulin [IVIG]), anticoagulants (LMWH), anti-inflammatory agents (acetylsalicylic acid [ASA; aspirin]), supplements (folic acid, Vitamin E, calcium), and other agents (human chorionic gonadotropin).

This study did not specifically collect data on maternal adverse events potentially related to HCQ (e.g., gastrointestinal symptoms, skin reactions, ophthalmic abnormalities); such information was only recorded incidentally in electronic medical records (e.g., patient complaints during routine prenatal visits) without systematic screening or classification.

### Exposure definition

Hydroxychloroquine (HCQ) exposure was defined as any oral administration of HCQ during pregnancy, based on prescription records from the hospital’s electronic medical system and patient follow-up interviews. Detailed exposure information included: time of initiation, defined as gestational week when HCQ treatment started (recorded as the first prescription date after confirmation of pregnancy); duration of use, defined as total number of weeks of HCQ administration during pregnancy (calculated as the interval between the first and last prescription dates, adjusted for missed doses reported by patients); dose (standard prescribed dose was 0.1 g twice daily [total 0.2 g/day]), and adherence, assessed by prescription refill rates and patient self-reports, with a rate of ≥80% (actual medication days/prescribed medication days) defined as “good adherence”.

Patients who initiated HCQ before pregnancy and continued use during pregnancy were classified as HCQ-exposed, while those with no HCQ prescription records or adherence <50% were classified as HCQ non-exposed.

### Outcomes

Primary outcomes were gestational week at delivery, with births at ≥ 37 weeks defined as term and births at < 37 weeks defined as preterm. Secondary outcomes included mid-to-late pregnancy loss and maternal and fetal complications, defined in accordance with national and international guidelines. Causes of mid-to-late pregnancy loss included late-term miscarriage, stillbirth, and fetal malformation. Maternal and fetal complications included gestational diabetes (diagnosed via oral glucose tolerance test with ≥1 abnormal value), premature rupture of membranes (rupture before 37 weeks of gestation), pregnancy-induced hypertension (PIH, systolic blood pressure ≥140 mmHg and/or diastolic blood pressure ≥90 mmHg after 20 weeks of gestation), placenta previa, thyroid disease, amniotic fluid reduction (maximum vertical pocket <2 cm or amniotic fluid index <5 cm). placental complications (defined as placental structural and functional abnormalities, such as placental abruption, placental infarction, placental insufficiency, and placental adhesion/implantation), umbilical cord abnormalities, fetal growth restriction/intrauterine growth retardation (FGR/IUGR, estimated fetal weight <10th percentile for gestational age), fetal malformation, and fetal distress (abnormal fetal heart rate pattern or fetal scalp blood pH <7.20).

### Statistical analysis

Statistical analyses were performed using R v 3.6.1 (R Project for Statistical Computing) with the MatchIt and tableone packages for propensity score matching (PSM) and baseline balance assessment. Normally distributed data were presented as means ± standard deviations (SDs), non-normally distributed data as medians [Q25, Q75], and categorical data as counts and percentages. Data were compared using Student’s t-tests, Kruskal-Wallis analysis of variance (ANOVA), Wilcoxon signed-rank tests, or χ² tests, as appropriate.

Univariable and multivariable logistic regression analyses were conducted to identify predictors of pregnancy outcomes. Several models (unadjusted, Model 1 [M1], Model 2 [M2]) were developed with different sets of covariables for adjustment, defined *a priori* based on clinical experience and a literature search. Results were reported as odds ratios (ORs) with 95% confidence intervals (CIs).

The unadjusted model only analyzed the crude association between HCQ exposure and outcomes. Model 1 was adjusted for “basic confounders”, defined as variables with established clinical relevance to pregnancy outcomes in RPL-related studies (age, BMI, recurrent spontaneous abortion, recurrent implantation failure, presence of antiphospholipid antibodies, UCTD or SS, and serological markers [homocysteine, D-dimer, platelet aggregation test (PAGT), fasting glucose, fasting insulin, folate, Vitamin B12, 25-hydroxyvitamin D, triglycerides and cholesterol (15, 25). The biochemical variables (PAGT, homocysteine, Vitamin B12) were selected based on their established relevance to the pathophysiology of RPL, the defining feature of our study cohort. Specifically, PAGT is a marker of platelet activation, which plays a critical role in thrombotic placental complications that are major contributors to RPL. Homocysteine elevation and Vitamin B12 deficiency are associated with impaired endothelial function and abnormal fetal-placental perfusion, both of which are well-documented risk factors for RPL and poor pregnancy outcomes in women with autoimmune diseases (26–29). These biochemical parameters may independently influence pregnancy outcomes (26–29). Therefore, adjusting for these variables was essential to exclude their potential confounding effects and to more accurately evaluate the independent association between HCQ use and the pregnancy outcomes of interest. Model 2 was adjusted for the same covariables and medication utilization (LMWH, IVIG, prednisone, and aspirin). These medications are commonly used in pregnant women with autoimmune diseases and may interact with HCQ or independently affect preterm birth risk (30, 31).

As a sensitivity analysis to strengthen the robustness and interpretability of our findings, the main outcome (preterm birth) and key secondary outcomes (maternal-fetal complications) were re-analyzed in a matched cohort using the same univariable and multivariable logistic regression models as the unmatched cohort. To address baseline imbalances (e.g., higher rate of >3 previous implantation failures in patients exposed to HCQ, p=0.034), 1:1 nearest-neighbor PSM was conducted with a caliper width of 0.2 SDs of the propensity score. Matching variables included age, pre-pregnancy BMI, number of previous miscarriages, autoimmune disease type (APS/UCTD/SS), antiphospholipid antibody status, and use of LMWH/prednisone. Post-matching balance was assessed via standardized differences (SD < 0.1 indicated adequate balance).

Subgroup analysis was pre-specified to explore potential heterogeneity in the effect of HCQ on pregnancy outcomes (focusing on preterm birth, the primary outcome, and key secondary outcomes including maternal-fetal complications) across different types of autoimmune disease (APS, UCTD, SS). These diseases exhibit distinct pathophysiological mechanisms (e.g., APS is characterized by antiphospholipid antibody-mediated coagulation disorders, UCTD presents with incomplete manifestations of connective tissue disease, and SS is associated with exocrine gland inflammation) and clinical management differs, which may lead to variations in patients’ response to HCQ. As these diseases are extremely common in outpatient clinics, this subgroup analysis should provide targeted references for treatment decision-making. For each subgroup (APS, UCTD, SS), the same univariable and multivariable logistic regression models (unadjusted, M1, M2) used in the overall analysis were applied to estimate the ORs and 95% CIs of the association between HCQ exposure and pregnancy outcomes. Heterogeneity across subgroups was assessed using Cochran’s Q test, with a two-sided p > 0.05 indicating no significant difference in HCQ’s effect across disease type.

Interaction analysis was performed to assess the impact of combining HCQ with prednisone on pregnancy outcomes.

A sensitivity analysis, varying sample size and statistical power, was performed to further assess the robustness of the findings.

*p* <.05 was considered statistically significant.

## Results

### Study population

Of 548 eligible patients, 56 were excluded due to loss to follow-up. Reasons included 1) lack of contact (27 cases, 48.2%): 19 cases due to relocation or transfer to other hospitals (without providing new contact information) and 8 cases due to invalid contact details or family refusal to cooperate; 2) voluntary withdrawal (15 cases, 26.8%): 9 cases due to intolerance to the frequency of serological testing and 6 cases due to concerns about privacy; 3) missing information on outcomes (14 cases, 25.0%): 10 cases of delivery at other hospitals without knowledge of outcomes and 4 cases of pregnancy termination (induced abortion or late miscarriage).

**Figure 1:**
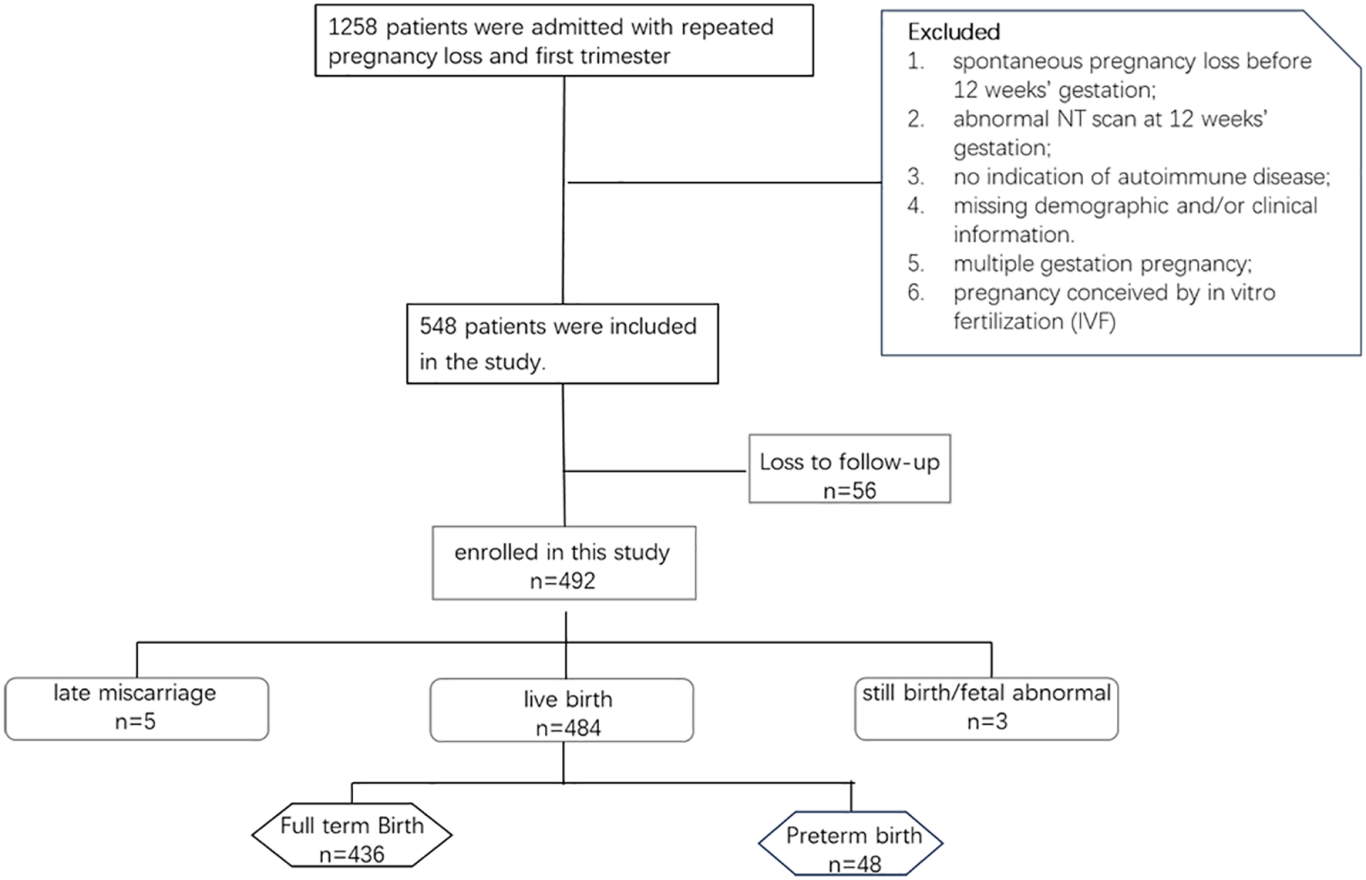
Study cohort.

Finally, 492 patients were included in the study cohort, and relevant data were extracted from electronic medical records for subsequent analysis; of these, 103 (20.9%) patients were exposed to HCQ, and 389 (79.1%) patients were not. The prescribed treatment regimen consisted of an oral administration of 0.1 g HCQ twice daily. The study flow chart of patient selection is shown in **Figure 1**.

Patient baseline demographic and clinical characteristics stratified by HCQ exposure are presented in **Table 1**. HCQ-exposed patients had a significantly higher number of > 3 previous implantation failures and were significantly more likely to have positive serology for SS vs. HCQ non-exposed patients. There were no significant differences in other demographic or clinical characteristics between HCQ-exposed vs. non-exposed patients.

**Table 1:** Baseline demographic and clinical characteristics of patients stratified by HCQ exposure.

Characteristic	HCQ non-exposure	HCQ exposure	*p* value
N	389	103	
Age, mean ± SD (years)	30.45 ± 3.65	31.13 ± 3.71	0.097
BMI, mean ± SD (kg/m^2^)	21.49 ± 2.66	21.80 ± 2.70	0.301
History of recurrent spontaneous abortion, n (%)			0.881
No	62 (15.94%)	17 (16.50%)	
Yes	327 (84.06%)	86 (83.50%)	
History of recurrent implantation failure, n (%)			0.497
No	345 (88.69%)	89 (86.41%)	
Yes	44 (11.31%)	14 (13.59%)	
Previous miscarriages, n (%)			0.216
2	190 (48.84%)	41 (39.81%)	
3	155 (39.85%)	46 (44.66%)	
>3	44 (11.31%)	16 (15.53%)	
Previous implantation failures, n (%)			**0.034**
2	359 (92.29%)	92 (89.32%)	
3	14 (3.60%)	1 (0.97%)	
>3	16 (4.11%)	10 (9.71%)	
Antiphospholipid antibody positive (anticardiolipin antibody, anti-β2-glycoprotein I antibody, lupus anticoagulant), n (%)			0.091
No	164 (42.16%)	53 (51.46%)	
Yes	225 (57.84%)	50 (48.54%)	
Undifferentiated connective tissue disease, n (%)			0.440
No	233 (59.90%)	66 (64.08%)	
Yes	156 (40.10%)	37 (35.92%)	
Sjögren's syndrome, n (%)			**0.023**
No	384 (98.71%)	98 (95.15%)	
Yes	5 (1.29%)	5 (4.85%)	
Serological testing, mean ± SD			
Homocysteine (µmol/l)	8.23 ± 3.66	8.18 ± 1.98	0.902
D-dimer (mg/l)	0.30 ± 1.25	0.27 ± 0.26	0.814
Platelet aggregation test (PAGT) (%)	66.46 ± 11.43	65.98 ± 11.96	0.715
Fasting glucose (mmol/l)	4.96 ± 0.41	4.93 ± 0.39	0.592
Postprandial glucose at 2 hours (mmol/l)	6.12 ± 1.34	6.36 ± 1.53	0.135
Fasting insulin (μIU/mL)	8.13 ± 4.39	7.78 ± 4.03	0.475
Postprandial insulin at 2 hours (μIU/mL)	56.68 ± 42.22	57.70 ± 45.21	0.836
Thyroid stimulating hormone (mIU/L)	2.18 ± 1.09	2.29 ± 1.49	0.402
Free thyroxine (T4) (pmol/L)	17.18 ± 2.81	16.25 ± 3.55	0.055
Folate (ng/ml)	16.71 ± 34.71	14.94 ± 5.39	0.617
Vitamin B12 (pg/ml)	460.98 ± 195.90	486.40 ± 213.86	0.266
25-hydroxyvitamin D (nmol/l)	43.84 ± 20.89	46.68 ± 32.74	0.299
Triglycerides (mmol/l)	1.64 ± 2.41	1.21 ± 0.97	0.135
Cholesterol (mmol/l)	4.16 ± 1.58	4.23 ± 1.02	0.709

N = sample size; mean ± SD = mean ± standard deviation; n (%) = number of cases (percentage of the corresponding group); Bold p-values indicate statistically significant differences (p < 0.05) between the hydroxychloroquine (HCQ) non-exposure and HCQ exposure groups.

### Pregnancy outcomes

Pregnancy outcomes stratified by HCQ exposure are presented in **Table 2**. There were 436 full-term births, 48 preterm births (28-36^+6^weeks), 5 late-term miscarriages (12-27^+6^weeks), 2 stillbirths (≥20 weeks), and 1 case of induced abortion due to fetal malformation. There were no significant differences in pregnancy outcomes between HCQ-exposed vs non-exposed patients.

**Table 2:** Pregnancy outcomes stratified by HCQ exposure.

Outcome	HCQ non-exposure	HCQ exposure	*p* value
Livebirth, n (%)			0.554
No	7 (1.80%)	1 (0.97%)	
Yes	382 (98.20%)	102 (99.03%)	
Preterm birth, n (%)			0.701
No	351 (90.00%)	94 (91.26%)	
Yes	39 (10.00%)	9 (8.74%)	
Late abortion, n (%)			0.961
No	386 (98.97%)	102 (99.03%)	
Yes	4 (1.03%)	1 (0.97%)	
Stillbirth, n (%)			0.466
No	388 (99.49%)	103 (100.00%)	
Yes	2 (0.51%)	0 (0.00%)	
Fetal abnormality, n (%)			0.607
No	389 (99.74%)	103 (100.00%)	
Yes	1 (0.26%)	0 (0.00%)	

As there are few studies on the relationship between HCQ exposure and preterm birth, and preterm birth is a major concern for obstetricians, the effect of HCQ on preterm birth was investigated. Univariable analysis of patients baseline demographic and clinical characteristics showed an association between postprandial glucose at 2 hours and preterm birth (OR 0.76 [95% CI 0.58, 0.99], *p* = 0.04), but no associations between patient age, pre-pregnancy BMI, other pre-pregnancy serology test results or medication utilization (**Table 3**).

**Table 3:** Univariable analysis of patients baseline demographic and clinical characteristics and preterm birth.

Characteristic	OR	95 L	95 H	P value
BMI, mean ± SD (kg/m^2^)	0.98	0.87	1.1	0.687
Age, mean ± SD (years)	1.01	0.93	1.1	0.754
Homocysteine (µmol/l)	0.96	0.83	1.11	0.57
D-dimer (mg/l)	0.97	0.68	1.39	0.878
Platelet aggregation test (PAGT) (%)	1.01	0.98	1.04	0.434
Fasting glucose (mmol/l)	1.17	0.55	2.49	0.685
Postprandial glucose at 2 hours (mmol/l)	**0.76**	**0.58**	**0.99**	**0.043**
Fasting insulin (μIU/mL)	1.04	0.97	1.11	0.246
Postprandial insulin at 2 hours (μIU/mL)	1	0.99	1.01	0.818
Vitamin B12 (pg/ml)	1	1	1	0.845
25-hydroxyvitamin D (nmol/l)	1	0.99	1.01	0.856
Acetylsalicylic acid-Yes	0.6	0.33	1.09	0.094
Prednisone-Yes	0.8	0.44	1.45	0.453
Low molecular weight heparin(LMWH)-Yes	0.77	0.43	1.4	0.399
Intravenous immunoglobulin(IVIG)-Yes	1.87	0.21	16.37	0.571
Hydroxychloroquine (HCQ)-Yes	0.86	0.4	1.84	0.701

OR = Odds Ratio; 95 L = 95% confidence interval (CI) lower bound; 95 H = 95% CI upper bound; p = statistical symbol for p-value (italicized per academic convention); bolded p-values indicate statistically significant results (p < 0.05).

Univariable and multivariable analyses showed a potential trend toward a protective effect of HCQ exposure on preterm birth, but no significant association (unadjusted multivariable model: OR 0.86 [95% CI 0.40, 1.84], *p* = 0.70; M1: OR 0.59 [95% CI 0.19, 1.84], *p* = 0.37; M2: OR 0.41 [95% CI 0.11, 1.51], *p* = 0.18) (**Table 4A**).The wide confidence intervals in the adjusted models reflect the limited statistical power to confirm this trend.

**Table 4A:** HCQ and preterm birth.

Exposure	Unadjusted	Model 1(Unmatched)	Model 2 (Unmatched)	Model 2 (PSM-matched)
HCQ				
No	1.0	1.0	1.0	1.0
Yes	0.86 (0.40, 1.84) *P* = 0.701	0.59 (0.19, 1.84) *P* = 0.365	0.41 (0.11, 1.51) *P* = 0.182	0.45 (0.12-1.71) P = 0.24

1.Model 1 (Unmatched) was adjusted for age, BMI, recurrent spontaneous abortion, recurrent implantation failure, presence of antiphospholipid antibodies, UCTD or SS, and serological markers (homocysteine, D-dimer, PAGT, fasting glucose, fasting insulin, folate, Vitamin B12, 25-hydroxyvitamin D, triglycerides and cholesterol).

2.Model 2 (Unmatched)was adjusted for the same covariables and medication utilization (LMWH, IVIG, prednisone, and aspirin).

3. Model 2 (PSM-matched) was based on a 1:1 propensity score-matched cohort (n=206, 103 per group), with adjustment for the same variables as Model 2 (Unmatched); matching variables included age, pre-pregnancy BMI, number of previous miscarriages, autoimmune disease type (APS/UCTD/SS), antiphospholipid antibody status, and use of LMWH/prednisone.

4. Data are presented as ORs with 95% CIs.

**Table 4B:** Subgroup analysis of HCQ exposure and preterm birth by type of autoimmune disease.

Subgroup	OR	95 L	95 H	P value
Antiphospholipid Syndrome (APS)	0.78	0.28	2.22	0.64
Undifferentiated Connective Tissue Disease (UCTD)	0.52	0.15	1.81	0.3
Sjögren's Syndrome (SS)	1.03	0.12	8.87	0.98

1. Heterogeneity test (Cochran’s Q test) showed no significant difference in the effect of HCQ on preterm birth across subgroups of patients with APS, UCTD, and SS (p=0.82). 2. Preterm birth rates were calculated as (preterm cases/subgroup total cases); e.g., the 20.00% rate in HCQ-exposed patients with SS reflects 1 preterm case among 5 exposed patients, with 0 cases in 5 non-exposed patients with SS. 3. The SS subgroup (n=10, 1 preterm case) had severely limited statistical power, with wide confidence intervals reflecting uncertainty in the estimate. This result is exploratory and not definitive; no conclusions regarding the effect of HCQ in patients with SS can be drawn from this subgroup.

HCQ and prednisone are commonly used in patients with autoimmune disease; therefore, the effectof HCQ plus prednisone on preterm birth was investigated. Univariable and multivariable analyses showed no significant differences in the incidence of preterm birth between prednisone-exposed (n=26 [54.17%]) vs. non-exposed (n=22 [43.85%]) patients (*p* = 0.537), and no significant associations between prednisone exposure (OR 0.84 [95%CI 0.43, 1.63], *p* = 0.612) or HCQ plus prednisone exposure (OR 0.72 [95% CI 0.29, 1.78], *p* = 0.479) and preterm birth (**Table 5**). Interaction analysis was performed to assess the impact of combining HCQ with prednisone on pregnancy outcomes. The p-value of the interaction analysis was 0.732, indicating that the analysis was underpowered (small sample size of combined medication users) and the results are not conclusive and should be interpreted with caution.

**Table 5:** HCQ and prednisone.

A. Incidence of preterm birth			
Preterm birth	No	Yes	*P*-value
HCQ			0.852
No	350 (78.83%)	39 (81.25%)	
Yes	94 (21.17%)	9 (18.75%)	
PRED			0.537
No	180 (40.22%)	22 (45.83%)	
Yes	266 (59.78%)	26 (54.17%)	
B. Interaction test			
Exposure	PRED	N	Crude
HCQ			
No	No	185	Ref.
Yes	No	16	0.86 (0.40, 1.84); P = 0.701
No	Yes	204	0.84 (0.43, 1.63); P = 0.612
Yes	Yes	87	0.72 (0.29, 1.78); P = 0.479
P interaction			0.732

Subgroup analysis stratified by major autoimmune diagnosis (APS, UCTD, SS), showed no significanteffect of HCQ on pregnancy outcomes between patients with APS (n=124) or UCTD (n=193) (p heterogeneity = 0.82). Due to the extremely small sample size (n=10) and only one preterm birth in patients with SS, statistical power was insufficient to determine the role of HCQ in patients with SS. No conclusions regarding the effect of HCQ in patients with SS can be drawn from this subgroup, and the results of this subgroup were not included in the overall heterogeneity assessment (**Table 4B**).

### Maternal and fetal complications

Fetal weight and maternal and fetal complications stratified by HCQ exposure are presented in**Table 6**. There were no significant differences in fetal weight or the incidence of maternal and fetal complications between HCQ-exposed vs non-exposed patients.

**Table 6:** Maternal and fetal complications of singleton pregnancies stratified by HCQ exposure.

Characteristic	HCQ non-exposure	HCQ exposure	*p* value
N	365	96	
Fetal weight, mean ± SD (g)	3226.96 ± 500.01	3215.46 ± 459.21	0.839
Apgar score, mean ± SD	9.50 ± 0.64	9.35 ± 0.74	0.172
Gestational diabetes, n (%)			0.832
No	299 (84.23%)	80 (83.33%)	
Yes	56 (15.77%)	16 (16.67%)	
Premature rupture of membranes, n (%)			0.459
No	339 (92.88%)	87(90.63%)	
Yes	26 (7.12%)	9 (9.37%)	
Pregnancy-induced hypertension, n (%)			0.104
No	348 (95.34%)	95 (98.96%)	
Yes	17 (4.66%)	1 (1.04%)	
Placenta previa, n (%)			0.854
No	344 (94.25%)	90 (93.75%)	
Yes	21 (5.75%)	6 (6.25%)	
Thyroid disease, n (%)			0.368
No	285 (78.08%)	79 (82.29%)	
Yes	80 (21.92%)	17 (17.71%)	
Amniotic fluid reduction, n (%)			0.055
No	349 (95.62%)	87 (90.63%)	
Yes	16 (4.38%)	9 (9.37%)	
Umbilical cord abnormality, n (%)			0.332
No	355 (97.26%)	95 (98.96%)	
Yes	10 (2.74%)	1 (1.04%)	
Fetal growth restriction/intrauterine growth restriction			0.351
No	359 (98.36%)	93 (96.88%)	
Yes	6 (1.64%)	3 (3.13%)	
Fetal malformation, n (%)			0.608
No	364 (99.73%)	96 (100.00%)	
Yes	1 (0.27%)	0 (0.00%)	
Placental complications, n (%)			0.917
No	358 (98.08%)	94 (97.92%)	
Yes	7 (1.92%)	2 (2.08%)	
Fetal distress, n (%)			0.200
No	341 (93.42%)	93 (96.88%)	
Yes	24 (6.58%)	3 (3.12%)	

Gestational diabetes mellitus (definition: ≥1 abnormal value in oral glucose tolerance test); premature rupture of membranes (definition: membrane rupture before 37 weeks of gestation); pregnancy-induced hypertension (definition: systolic blood pressure ≥140 mmHg and/or diastolic blood pressure ≥90 mmHg after 20 weeks of gestation); amniotic fluid reduction (definition: maximum vertical pocket of amniotic fluid <2 cm or amniotic fluid index <5 cm); fetal distress (definition: abnormal fetal heart rate, monitoring or fetal scalp blood pH <7.20); placental complications (conditions related to placental structural and functional abnormalities, such as placental abruption, placental infarction, placental insufficiency, and placental adhesion/implantation).

### Sensitivity analysis: propensity score-matched cohort

To address baseline imbalances (e.g., higher rate of >3 previous implantation failures and SS seropositivity in HCQ-exposed patients), 1:1 PSM was performed. Post-matching baseline characteristics of HCQ-exposed and non-exposed patients are presented in **Supplementary Table S1**. After matching, all variables showed standardized differences < 0.1, indicating adequate balance between the two patient groups. Specifically, the difference in the rate of >3 previous implantation failures (from 0.21 pre-matching to 0.07 post-matching) and SS seropositivity (from 0.15 pre-matching to 0.08 post-matching) were eliminated. Demographic characteristics, autoimmune disease distribution, serological markers, and medication use were also well-balanced, ensuring that the sensitivity analysis of pregnancy outcomes and maternal and fetal complications were not confounded by differences at baseline.

After 1:1 PSM, 206 patients (103 HCQ-exposed vs. 103 non-exposed) were included in the sensitivity analysis. Consistent with the unmatched cohort, multivariable logistic regression in the matched cohort showed no statistically significant association between HCQ exposure and preterm birth (OR 0.52 [95% CI 0.16, 1.68], p=0.27; Model 2 adjusted for medications: OR 0.45 [95% CI 0.12, 1.71], p=0.24), retaining the potential protective trend observed in the main analysis.

For secondary outcomes, no significant differences were found in fetal weight (3208.7 ± 462.3 g vs. 3231.2 ± 498.5 g, p=0.81) or maternal-fetal complication rates (e.g., gestational diabetes: 8.7% vs. 10.7%, p=0.68; pregnancy-induced hypertension: 1.0% vs. 3.9%, p=0.32) between HCQ-exposed vs non-exposed patients in the matched cohort. These results confirm that the primary findings were not confounded by baseline imbalances, supporting the robustness of the study conclusions (**Table 4A**, **Supplementary Table S1**).

### Sensitivity analysis: sample size and statistical power

**Figure 2:**
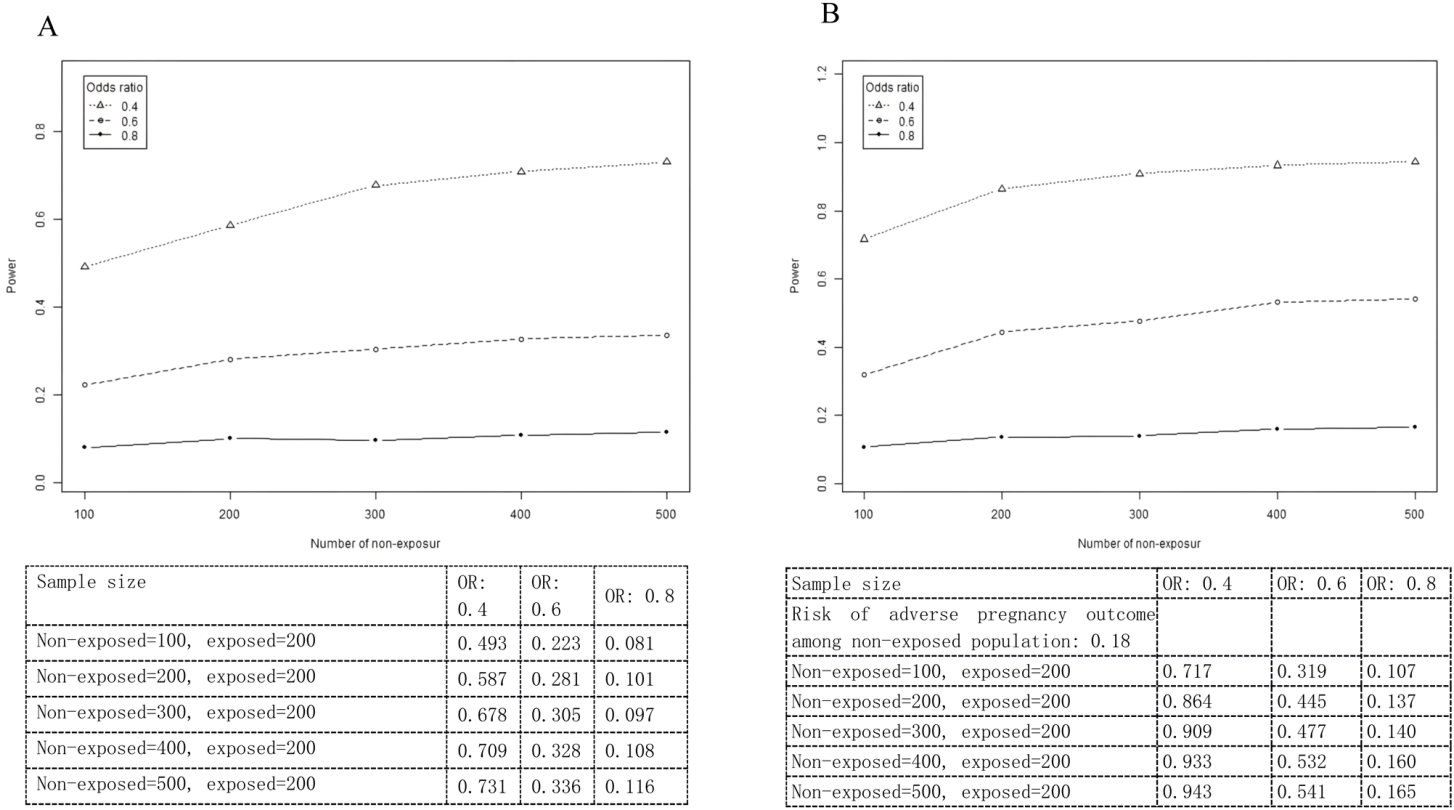
Sensitivity analysis. **(A)** Risk of adverse pregnancy outcomes among the non-exposed population: 0.1. **(B)** Risk of adverse pregnancy outcomes among the non-exposed population: 0.18.

Sensitivity analysis was also conducted to explore the effect of power and sample size on study results. Findings showed that even when the sample size of non-exposed patients increased, the association between HCQ exposure and adverse pregnancy outcomes did not reach significance, suggesting the results of this study are robust (**Figure 2**). Combined with the consistent results from the PSM-matched cohort analysis, these findings further confirm the robustness of the study conclusions.

## Discussion

This study explored the effects of HCQ on pregnancy outcomes in women with a history of RPL and autoimmune disease, with a particular focus on gestational age at delivery and maternal and fetal complications. Findings showed that HCQ was not associated with an increased risk of preterm birth, late abortion, stillbirth, or maternal or fetal complications (including gestational diabetes, PIH, placenta previa, placental complications, FGR/IUGR, fetal malformation and fetal distress). A potential protective trend against preterm birth was observed, but this association did not reach statistical significance. These results support the safety of HCQ in this high-risk population, while the observed protective trend requires confirmation in larger studies.

To the authors’ knowledge, this is the first study to focus on the effects of HCQ on pregnancy outcomes in women with a history of RPL and various autoimmune diseases, including in China. Most previous studies have focused on the effects of HCQ in patients with APS, a leading cause of RPL (25), and some have characterized pregnancy complications and treatment in obstetric APS in China (32, 33).

Consistent with the multivariable analysis results (Model 2 OR 0.41, p=0.18), the potential protective trend of HCQ on preterm birth may be related to its anti-inflammatory and vascular-protective effects [inhibiting placental inflammation and improving trophoblast function (18)]. Notably, the median gestational week of HCQ initiation in our study was 12 weeks (second trimester), when placental development is already established. This timing may allow HCQ to target placental inflammation without interfering with early embryonic development, which could explain why our results differ from studies reporting teratogenic risks with first-trimester HCQ exposure. These observations need to be confirmed by larger sample studies.

Pregnancies in patients with autoimmune disease are challenging to manage due to autoimmune disease activity, the presence of some autoantibodies, pharmacological therapy, comorbidities, and the potential for adverse maternal and fetal outcomes (34). Pregnancy may increase disease activity in patients with some autoimmune diseases, and exacerbation of disease could be detrimental to both mother and fetus. While teratogenic drugs should be discontinued before conception in patients with autoimmune disease planning pregnancy, evidence suggests that it is beneficial to continue HCQ throughout gestation (34).

HCQ has dual immunomodulating and vascular protective effects. Specifically, HCQ inhibits antigen processing and presentation, subsequent T-cell activation, and the production of pro-inflammatory cytokines. It has vascular protective effects through the reduction of endothelial dysfunction and hypercoagulability, particularly through the inhibition of antiphospholipid antibody binding and platelet aggregation.

While there is consensus HCQ should be maintained throughout pregnancy in women with autoimmune disease, the safety of HCQ is still debated. Previous studies showed that first-trimester exposure to HCQ among individuals with SLE or RA was not associated with a significantly increased risk of major congenital malformations (23, 35) and there were no differences in live birth rates, preterm birth rates, or adverse pregnancy outcomes such as miscarriage, premature rupture of membranes or neonatal morbidity in women with various connective tissue diseases (36) or SLE (37, 38) exposed to vs. not exposed to HCQ during pregnancy. However, in one meta‐analysis HCQ exposure was associated with an increased rate of spontaneous abortion in women with autoimmune disease. The present study adds to this body of evidence showing that HCQ did not increase the risk of preterm birth, late abortion, stillbirth, or maternal or fetal complications in women with a history of RPL and autoimmune disease. Notably, our study focused on women with both RPL and autoimmune disease, a population at higher pregnancy risk than those with autoimmune disease alone. The safety of HCQ in this specific population further supports its clinical application.

In the context of RPL, the immunomodulatory, anti-thrombotic, vascular-protective, and anti-infectious effects of HCQ may be protective. Dysregulation of the maternal immune system is an underlying mechanism of RPL, as the balance between immune activation and tolerance within the endometrium is essential for successful pregnancy. HCQ may prevent an overactive pro-inflammatory response at the maternal–fetal interface and promote the vascular remodeling of spiral arteries essential for placentation (15). A systematic review showed HCQ in addition to aspirin and heparin had a significant benefit for mitigating the risk of antiphospholipid antibody-mediated obstetrical complications (39) (live birth rate, pregnancy loss, maternal complications, neonatal complications) in patients with APS. A double-blind placebo-controlled trial investigating the effect of HCQ on pregnancy outcomes in unexplained RPL showed the prevalence of abortion in individuals exposed to HCQ was four times lower than in individuals exposed to placebo, however, this difference was not statistically significant (40). In the present study, there was no significant difference in the number of live births in women with a history of RPL and autoimmune disease exposed to vs. not exposed to HCQ implying HCQ had limited impact on the mechanisms underlying RPL in this patient population.

While our study focused on pregnancy and fetal outcomes, it is important to acknowledge that we did not systematically assess HCQ-related maternal adverse events. Previous studies have reported mild, transient side effects of HCQ in pregnant women (e.g., gastrointestinal symptoms, skin reactions) that rarely require treatment discontinuation (35, 36). Given the lack of systematic screening in our study, we cannot rule out the possibility of underreported mild maternal adverse effects; however, no severe adverse events (e.g., retinal toxicity, severe hematological abnormalities) were incidentally recorded in medical records, which is consistent with the overall favorable safety profile of HCQ reported elsewhere (41–44).

Notably, treatment options for pregnant women with autoimmune diseases are evolving. One study showed that HCQ combined with low-dose aspirin was associated with a higher proportion of full-term pregnancies and a significantly lower proportion of hypertension, prematurity, and pregnancy loss than HCQ alone in women with SLE. A multicenter study exploring the effects of additional treatments combined with conventional therapies in pregnant patients with high-risk APS suggests that preconception HCQ therapy significantly reduces the risk of fetal death in cases previously refractory to low-dose aspirin and heparin alone, offering a promising strategy for improving pregnancy outcomes in high-risk patients with APS (45). Future studies should focus on the dosage of HCQ, its duration of use, and its safety when used in combination with other immunosuppressive medications in pregnant women with autoimmune diseases.

### Limitations

Although this study provides important clinical evidence for the use of HCQ in women with a history of RPL and autoimmune diseases, it has certain limitations that should be acknowledged when interpreting the results. First, the sample size of this study was relatively small, which may affect the external validity of the results. Second, the study primarily focused on mid-to-late pregnancy outcomes and lacked observations on the effect of HCQ use during the early stages of pregnancy. Third, the effects of HCQ may differ for pregnant women with different types of autoimmune diseases, and future research should involve more detailed subgroup analyses based on specific diseases. Fifth, the timing of HCQ initiation varied considerably among participants in our cohort, which may introduce residual confounding given that the potential effects of HCQ on pregnancy outcomes could be time-dependent. Sixth, HCQ adherence was assessed based on medical record documentation and patient self-report rather than objective measures (e.g., serum drug concentration testing or electronic medication monitoring). This subjective assessment method may lead to misclassification of adherence status, which could bias the observed associations between HCQ use and study outcomes. Seventh, multiple comparisons were conducted across various pregnancy outcomes, subgroup analyses, and interaction analyses in this study. Without formal statistical correction for multiple testing, the risk of type I error may be elevated, particularly for exploratory findings. Eighth, this study did not systematically collect data on HCQ-related maternal adverse events, which is a key consideration for drug safety evaluation in long-term use. Mild or asymptomatic maternal side effects (e.g., mild nausea, transient pruritus, subclinical ophthalmic changes) may have been overlooked during routine prenatal visits, as maternal adverse event screening was not a predefined study objective. Routine ophthalmic examinations (e.g., optical coherence tomography) targeting HCQ-related retinal toxicity were not performed for all exposed patients, further increasing the risk of under detecting subclinical maternal adverse effects. Last, the single-center observational design of this study prevents the establishment of causal relationships between HCQ use and pregnancy outcomes.

These limitations highlight the need for future studies to fully evaluate the use of HCQ in pregnant women with a history of RPL and autoimmune diseases.

## Conclusions

This study suggests that HCQ use in pregnant women with a history of RPL and autoimmune diseases is not associated with an increased risk of preterm birth, or maternal or fetal complications, and may offer a protective effect. HCQ appears to be safe for maternal and fetal health during pregnancy, providing positive clinical evidence for managing pregnancies in high-risk patients. Due to the small sample size and retrospective design, the potential protective effect of HCQ on preterm birth needs to be confirmed by prospective multicenter studies with larger samples. Future research should also focus on long-term neonatal safety and the role of HCQ in different types of autoimmune disease.

## Data Availability

The original contributions presented in the study are included in the article/**Supplementary Material**. Further inquiries can be directed to the corresponding author.
