# Supplementary material for: Hydroxychloroquine therapy for women with both recurrent pregnancy loss and autoimmune disease: association with pregnancy outcomes and maternal-fetal complications
Source: Front Immunol. 2026 Feb 10;17:1756063. doi: 10.3389/fimmu.2026.1756063 (PMC12929442; doi:10.3389/fimmu.2026.1756063)
Supplement: Supplementary file 1 [file Table1.docx]

**Table S1 Baseline characteristics of the study population after propensity score matching (PSM)**

| Characteristic | HCQ Non-exposed (n=103) | HCQ Exposed (n=103) | Pre-matching Standardized Difference | Post-matching Standardized Difference |
| --- | --- | --- | --- | --- |
| Demographic Characteristics |  |  |  |  |
| Age, mean ± SD (years) | 30.62 ± 3.58 | 31.13 ± 3.71 | 0.14 | 0.08 |
| Pre-pregnancy BMI, mean ± SD (kg/m²) | 21.55 ± 2.61 | 21.80 ± 2.70 | 0.09 | 0.06 |
| Reproductive History |  |  |  |  |
| Previous miscarriages, n (%) |  |  | 0.12 | 0.05 |
| - 2 times | 50 (48.54) | 41 (39.81) | - | - |
| - 3 times | 39 (37.86) | 46 (44.66) | - | - |
| - >3 times | 14 (13.59) | 16 (15.53) | - | - |
| Previous implantation failures >3 times, n (%) | 4 (3.88) | 10 (9.71) | 0.21 | 0.07 |
| Autoimmune Disease-Related Indicators |  |  |  |  |
| Antiphospholipid antibody positive, n (%) | 59 (57.28) | 50 (48.54) | 0.18 | 0.09 |
| Autoimmune disease type, n (%) |  |  | 0.15 | 0.08 |
| - Antiphospholipid syndrome (APS) | 61 (59.22) | 62 (60.19) | - | - |
| - Undifferentiated connective tissue disease (UCTD) | 36 (34.95) | 37 (35.92) | - | - |
| - Sjögren's syndrome (SS) | 6 (5.83) | 4 (3.88) | - | - |
| Serological Marker |  |  |  |  |
| Postprandial glucose at 2 hours, mean ± SD (mmol/L) | 6.18 ± 1.31 | 6.36 ± 1.53 | 0.12 | 0.07 |
| Medication Use During Pregnancy |  |  |  |  |
| Use of low molecular weight heparin (LMWH), n (%) | 59 (57.28) | 61 (59.22) | 0.04 | 0.03 |
| Use of prednisone, n (%) | 61 (59.22) | 58 (56.31) | 0.06 | 0.05 |

Note: BMI = body mass index; SD = standardized difference. A standardized difference < 0.1 indicates adequate balance between HCQ-exposed and non-exposed patients after PSM. Data are presented as mean ± SD for continuous variables and n (%) for categorical variables.
